# Data Mining-Based Analysis of Chinese Medicinal Herb Formulae in Chronic Kidney Disease Treatment

**DOI:** 10.1155/2020/9719872

**Published:** 2020-01-24

**Authors:** Ping Xia, Kun Gao, Jiadong Xie, Wei Sun, Ming Shi, Wei Li, Jing Zhao, Jin Yan, Qiong Liu, Min Zheng, Xin Wang, Qijing Wu, Enchao Zhou, Jihong Chen, Lingdong Xv, Weiming He

**Affiliations:** ^1^Division of Nephrology, Affiliated Hospital of Nanjing University of Chinese Medicine, Jiangsu Province Hospital of Chinese Medicine, Nanjing 210029, China; ^2^School of Artificial Intelligence and Information Technology, Nanjing University of Chinese Medicine, Nanjing 210029, China; ^3^Division of Gerontology, The Third Affiliated Hospital of Nanjing University of Chinese Medicine, Nanjing 210000, China; ^4^Division of Nephrology, Suzhou Hospital of Integrated Medicine, Suzhou 215200, China

## Abstract

**Background:**

Traditional Chinese medicine (TCM) has long been used to treat chronic kidney disease (CKD) in Asia. Its effectiveness and safety for CKD treatment have been confirmed in documented studies. However, the prescription rule of formulae for Chinese medicinal herbs is complicated and remains uncharacterized. Thus, we used data mining technology to evaluate the treatment principle and coprescription pattern of these formulae in CKD TCM treatment.

**Methods:**

Data on patients with CKD were obtained from the outpatient system of a TCM hospital. We established a Chinese herb knowledge base based on the Chinese Pharmacopoeia and the Chinese Materia Medica. Then, following extraction of prescription information, we deweighted and standardized each prescribed herb according to the knowledge base to establish a database of CKD treatment formulae. We analyzed the frequency with which individual herbs were prescribed, as well as their properties, tastes, meridian tropisms, and categories. Then, we evaluated coprescription patterns and assessed medication rules by performing association rule learning, cluster analysis, and complex network analysis.

**Results:**

We retrospectively analyzed 299 prescriptions of 166 patients with CKD receiving TCM treatment. The most frequently prescribed core herbs for CKD treatment were Rhizoma Dioscoreae (Shanyao), Spreading Hedyotis Herb (Baihuasheshecao), Root of Snow of June (Baimagu), Radix Astragali (Huangqi), Poria (Fulin), Rhizoma Atractylodis Macrocephalae (Baizhu), Radix Pseudostellariae (Taizishen), and Fructus Corni (Shanzhuyu). The TCM properties of the herbs were mainly being warm, mild, and cold. The tastes of the herbs were mainly sweet, followed by bitter. The main meridian tropisms were Spleen Meridian of Foot-Taiyin, Liver Meridian of Foot-Jueyi, Lung Meridian of Hand-Taiyin, Stomach Meridian of Foot-Yangming, and Kidney Meridian of Foot-Shaoyin. The top three categories were deficiency-tonifying, heat-clearing, and dampness-draining diuretic.

**Conclusion:**

Using an integrated analysis method, we confirmed that the primary TCM pathogeneses of kidney disease were deficiency and dampness-heat. The primary treatment principles were tonifying deficiency and eliminating dampness-heat.

## 1. Introduction

Chronic kidney disease (CKD) is a worldwide public health problem and its prevalence is more than 10%. It eventually progresses to end-stage renal disease in 20–30% of cases, resulting in tremendous social and economic burdens [1, 2]. According to the Global Burden of Disease Study, CKD was the 19th leading cause of years of life lost in 1990 and rose to 16th place in 2016 and is expected to further rise to 5th place by 2040 [3]. How to slow or stop the progression of CKD is a major challenge for nephrologists. Unfortunately, early detection and effective treatment remain difficult to date. In China, the prevalence of CKD is 10.8% [4]. Many Chinese patients with CKD benefit from traditional Chinese medicine (TCM) [5], which is perceived as a cost-effective alternative medicine. TCM originated in ancient China and has continued to evolve for over 2,500 years. Notably, it has long been used to treat kidney disease [6]. The effectiveness and safety of TCM have progressed considerably over several decades, especially in the case of some herbs and herbal compounds prescribed for CKD treatment [7–10].

Syndrome differentiation and treatment are the core of TCM in clinical practice. There are three basic principles for curing disease with Chinese medicinal herbs: eliminating the cause of illness, dispelling pathogenic factors, and restoring coordination of the internal organs so that excess and deficient yin or yang can be corrected. Most TCM prescriptions consist of more than two herbs. According to the TCM theory, several herbs prescribed together are superior to a single herb for disease treatment because the full extent of their advantages is realized and their disadvantages are inhibited. Different therapeutic effects may result from prescribing different herbs and herb combinations; the toxicity of other coprescribed herbs may also be decreased. In clinical practice, it is necessary to be flexible in modifying the formulae for Chinese medicinal herbs according to the condition of the illness and the status of the patient under the guidance of TCM compatibility principles. The specific prescription reflects both the patient's characteristics and the physician's experience. Only when the principles are unified flexibly can the prescription be congruent with the syndromes to be treated. Then, the expected efficacy will be achieved. Thus, it is necessary to follow the principle but not adhere to the initially established formula. Collection and analysis of formulae are one of the essential methods for learning and passing on knowledge of TCM. However, flexible intervention is extremely complicated in clinical practice, which causes considerable difficulties for clinical research. It also limits TCM from further developing and becoming more widespread. Many researchers and nephrologists are concerned about how to utilize TCM herb formulae safely and effectively in kidney disease. Much attention has been paid to exploring the core herbs used in TCM, fundamental therapy principles, and prescription methods. Therefore, interpretation of herbal formulae is expected to help identify the most effective herbs and improve the treatment of kidney disease. It should also contribute to the development of novel drugs for CKD treatment.

Machine learning has been used to analyze the prescription patterns of Chinese medicine from clinical data and to discover potential associations between Chinese medicinal herbs and disease [11]. At present, data mining in the field of TCM covers a wide range of conditions, prescriptions, cases, and diagnostics. Frequency statistics, association rule learning, cluster analysis, and complex network analysis are commonly used techniques [12–14]. Scholars have surveyed clinical data from the National Health Insurance Database and used frequency analysis and association rule learning to explore TCM treatment for insomnia and depression. They showed the prescription patterns of Chinese herbs for patients with sleep disorder and major depressive disorder [12]. Another study was based on hierarchical clustering of herbal effects and determined standard prescription rules combined with the theory of qi and blood to explore the consistency of TCM theory and herbs [13]. We previously designed a prescription recommendation algorithm based on the complex network method to explore the core herbs for lung cancer treatment [14]. It is important to note complex factors of TCM which do not meet the requirements of data mining: clinical diagnosis and treatment information, various data structures, and high information dimensions. Therefore, it is necessary to determine a method that includes data cleansing and data mining.

In this study, we proposed an integrated analysis method to extract and mine TCM medical records. We collected clinical cases in which patients were given herbal prescriptions by one of our hospital's TCM specialists, Professor Lijuan Gong. Then, we analyzed prescriptions for CKD treatment using association rule learning, cluster analysis, and complex networks to explore conventional TCM prescriptions, the law of medication, and potential core prescriptions. We aimed to identify the main treatment principle and herb prescriptions of TCM specialists for CKD treatment using modern data computer technology.

## 2. Materials and Methods

### 2.1. Data Source

**Figure 1:**
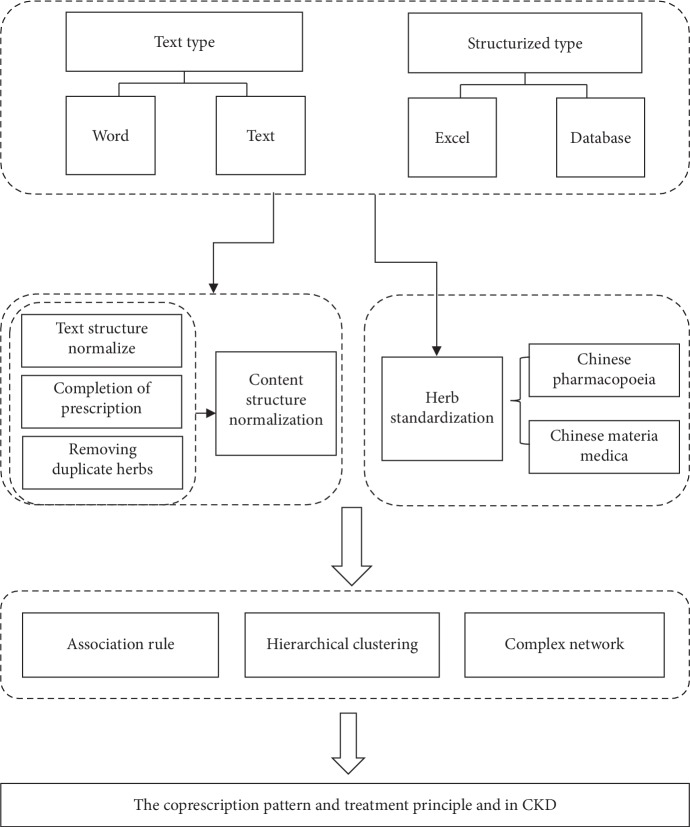
Data mining flowchart. The integrated data mining method included data processing, frequency statistics, association rules, cluster analysis, and complex network analysis.

We retrospectively analyzed a patient sample from the Jiangsu Province Hospital of Chinese Medicine and determined the prevalence of prescribed Chinese medicinal herbs in patients with CKD from 2011 to 2016. CKD diagnosis was based on the National Kidney Foundation Kidney Disease Outcome Quality Initiative 2002 guidelines and was defined as abnormalities in kidney structure or function present for >3 months or glomerular filtration rate <60 ml/min/1.73 m^2^. Exclusion criteria were (1) acute kidney injury diagnosis, (2) receiving renal replacement therapy, (3) incomplete prescription composition or dosage, and (4) not receiving herb formula treatment. In total, 299 prescriptions in 166 patients were enrolled in this study. The study procedures were approved by the Research Ethics Committee of the Affiliated Hospital of Nanjing University of Chinese Medicine and performed in strict conformity with our institutional guidelines. The detailed workflow diagram is summarized in Figure 1.

### 2.2. Data Processing

#### 2.2.1. Data Collection

We screened all the clinical information from the clinical cases of outpatients. Patient information was collected, including age, sex, home address, diagnosis, and TCM diagnosis. Herbal prescriptions at each patient's first visit and subsequent follow-up visits were recorded.

#### 2.2.2. Data Process

The data processing methods mainly included establishing the TCM knowledge base, normalizing the prescription's content structure, separating the compound medicine into individual herbs, standardizing TCM terminology, and correcting for manual error.

In brief, we first established a Chinese medicinal herb knowledge base according to the Chinese Pharmacopoeia and the Chinese Materia Medica [15, 16]. First, we identified the herbs in the prescriptions and unified them according to content structure based on the knowledge base. Second, we eliminated duplicate herbs and applied standardized terminology for the expression of the herbs. Then, we extracted information on prescriptions and herbs. All these data were transferred to Microsoft Excel 2016 to establish a TCM nephrology database. The information collection process was confirmed by two researchers.

### 2.3. Statistical Analysis

All the data were processed by R-Studio Version 3.5.3. First, we used the Apriori algorithm to analyze the association rules of the herbs. Second, hierarchical clustering was used to classify high-frequency herbs. Finally, complex network analysis was used to confirm the core herbs prescribed for CKD treatment.

#### 2.3.1. Apriori Algorithm

The Apriori algorithm is a frequent itemset algorithm for mining association rules. We used it to illustrate the specific rules of TCM in CKD treatment. In our data, each herb was treated as a variable. A fixed combination can be understood as a herb pair in TCM theory. The formulae were as follows:

(1) $$\begin{array}{c} \text{support}\left(X\;⟶\;Y\right)=\;\sigma \frac{\left(X\cup Y\right)}{N}\;,\\ \text{confidence}\left(X\;⟶\;Y\right)=\sigma \frac{\left(X\cup Y\right)}{\sigma \left(X\right)},\\ \text{lift}\left(X\;⟶\;Y\right)=\text{confidence}\frac{\left(X\;⟶\;Y\right)}{\sigma \left(Y\right)}, \end{array}$$

where *X* ⟶ *Y* is an association rule, *X* (left-hand side [LHS]) and Y (right-hand side [RHS]) represent the set of herb items, *σ*(*X*) is the frequency of itemset *X*, *X* ∪ *Y* is the union of itemset *X* and *Y*, *σ*(*X* ∪ *Y*) is the frequency with which itemset *X* and itemset *Y* appear together, support(*X* ⟶ *Y*) is the frequency with which *X* and *Y* appear together, and confidence(*X* ⟶ *Y*) is the probability that itemset *Y* appears in the presence of *X*. The lift is the ratio of the probability of itemset *Y* appearing in the presence of *X* to the frequency of *Y*. Support and confidence are often used to eliminate meaningless combinations; lift is the validity of the rules.

#### 2.3.2. Hierarchical Clustering Algorithm

In the hierarchical clustering algorithm, each herb was regarded as a cluster, and N clusters were combined to form a new class based on a similarity measure between objects. The Euclidean metric was used to calculate the similarity between herbs, the formula of which is

(2) $$\begin{array}{c} d\left(x,y\right)=\sqrt{\overset{n}{\underset{k=1}{\sum }}{\left(x_{k}-y_{k}\right)}^{2}}. \end{array}$$

Herbs used more than 30 times were extracted for word clustering.

#### 2.3.3. Complex Network Analysis

We used complex network analysis to confirm the core herb in each prescription. Complex network analysis is used to analyze complex interaction laws in complex systems in the real world based on a network model of nodes and edges. The complexities of diseases and human life systems are gradually being recognized such that medical research from a network perspective has become an important topic in current medical research. We regarded the constituent herbs for CKD treatment as nodes and connections between two herbs as edges. Thus, we could rationalize all medical record data into a network of drug nodes and edges using Liquorice (a complex network analysis tool) and the multiscale backbone algorithm.

Node degree and confidence level are the keys to measuring the association between drugs. Node degree is the number of edges connected to a node, and the confidence level represents the reliability of the data. The multiscale backbone is a statistical model established by identifying significant edges; the parts of the network that are retained are those in which the nodes are strongly associated. We set the screening criteria as a node degree of 36 and a confidence level of 0.95.

## 3. Results

### 3.1. CKD Patient Characteristics

We analyzed 299 prescriptions of 166 patients with CKD. Of the patients, 66 (39.7%) were male and 100 (60.3%) were female, with a male to female ratio of 1 : 1.51. Age ranged from 4 to 82 (mean, 48.92 ± 17.69) years. On the kidney disease spectrum, most patients had glomerular disease, hypertensive kidney disease, or diabetic kidney disease.

### 3.2. Herb Frequency and Analysis

We analyzed herb frequency in all prescriptions because formulae for Chinese medicinal herbs usually consider two or more herbs. Among the 299 prescriptions, 202 Chinese medicinal herbs appeared 4123 times, as shown in Table 1. The most frequently prescribed herb was Rhizoma Dioscoreae (Shanyao), which appeared 231 times (frequency, 77.3%). The following most frequently prescribed herbs in descending order were Spreading Hedyotis Herb (Baihuasheshecao), Root of Snow of June (Baimagu), Radix Astragali (Huangqi), Poria (Fulin), Rhizoma Atractylodis Macrocephalae (Baizhu), Radix Pseudostellariae (Taizishen), Fructus Corni (Shanzhuyu), Radix Rehmanniae (Shengdihuang), Fructus Lycii (Gouqizi), Flos Carthami (Honghua), Common Sage Herb (Lizhicao), Pericarpium Citri Reticulate (Chenpi), Cortex Phellodendri (Huangbai), Semen Coicis (Yiyiren), Rhizoma Imperatae (Baimaogen), and Rhizoma Anemarrhenae (Zhimu). These top 17 most frequently prescribed herbs appeared more than 78 times (frequency, more than 25%). These results demonstrate that these were preferred herbs in CKD.

### 3.3. Properties, Tastes, and Meridian Tropisms of Herbs

**Figure 2:**
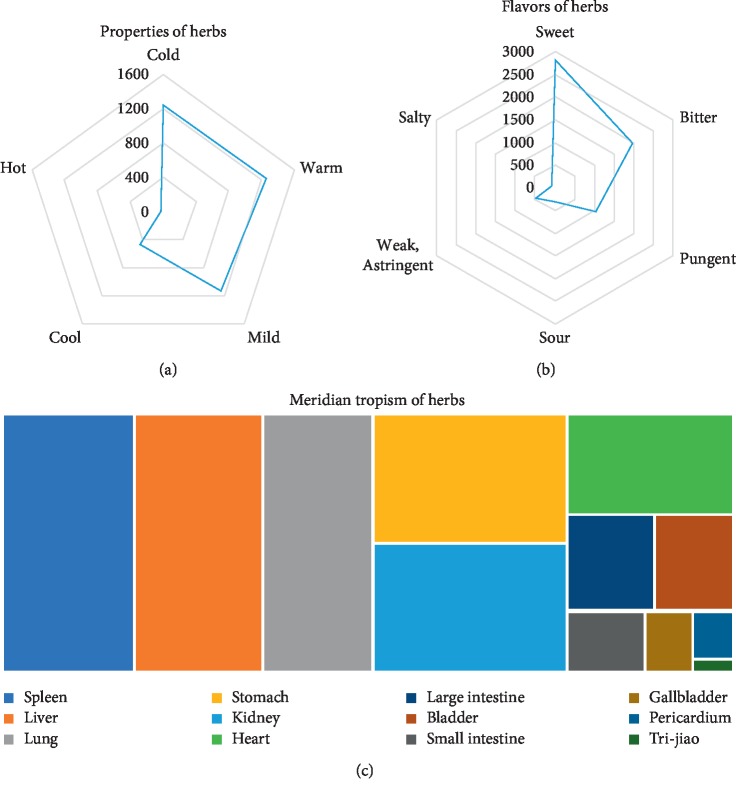
Property, taste, and meridian tropism of herbs. (a) Herb properties. All herbs in each prescription were analyzed using a radar chart divided into five categories. (b) Herb taste. Tastes were divided into six categories using a radar chart. (c) Meridian tropism of herbs. We created a tree diagram of the meridian tropism of all herbs. Different meridian tropisms are indicated by different colors as shown at the bottom of the diagram. All images were analyzed using Microsoft Excel 2016.

We analyzed the properties and tastes of the herbs. As Figure 2(a) shows, the TCM properties were mainly being warm, mild, and cold. We adopted five tastes for analysis: pungent, sweet, sour, bitter, and salty. The herbs mainly tasted sweet, followed by bitter (Figure 2(b)). Sweet taste has nourishing, harmonizing, and moistening functions. Bitter taste has the functions of clearing away dampness and purging. These data indicate that nourishing and purging were the principal functions of TCM in CKD treatment. Meridian tropism refers to medicinal herbs that often and selectively produce therapeutic effects on specific parts of the human body. The theory of meridian tropism plays a vital role in the clinical selection of Chinese medicinal herbs. The frequency of a herb's meridian tropism can be determined by classification using a tree diagram: Spleen Meridian of Foot-Taiyin, Liver Meridian of Foot-Jueyi, Lung Meridian of Hand-Taiyin, Stomach Meridian of Foot-Yangming, and Kidney Meridian of Foot-Shaoyin. Our results showed that therapeutic effects were predominant for the kidney, spleen, liver, and lung and reflected that TCM is holistic.

### 3.4. Action Category of Herbs

Chinese medicinal herbs have specific efficacy and can be classified into different specific action categories.  Table 2 details the categories and the frequency with which herbs were classified into these categories. The top ten categories for CKD treatment were deficiency-tonifying herbs, heat-clearing herbs, dampness-draining diuretic herbs, hemostatic herbs, astringent herbs, qi-regulating herbs, blood-activating and stasis-resolving herbs, cough-suppressing and panting-calming herbs, exterior-releasing herbs, and digestant herbs.

### 3.5. Prescription Patterns of Chinese Medicinal Herbs

Compatibility of Chinese medicinal herbs refers to the combination of two or more herbs with a purpose based on clinical requirements and medicinal properties and actions. It is the primary clinical method of medicinal application and the basis of herbal formulae composition. Here, we used data mining to assess the standard prescription patterns of the formulae for CKD treatment.

#### 3.5.1. Herb Pair Analysis

In clinical practice, paired usage of Chinese medicinal herbs is essential therapeutically and comprises the herb prescription method. Thus, we analyzed herb pairs.

**Figure 3:**
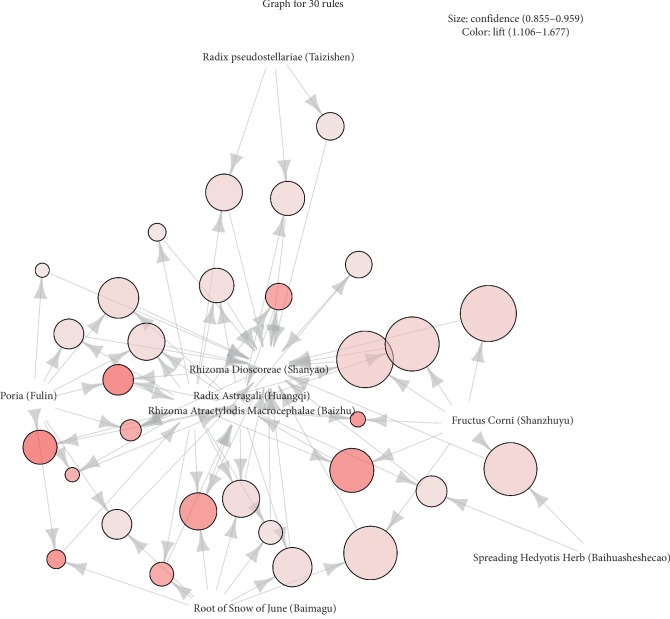
Association rule combination matrix. The association rule combination matrix was analyzed using R-studio 3.5.3. Size indicatesconfidence (0.855–0.959) and color indicates lift (1.106–1.667).

First, we used the Apriori algorithm to analyze the association rules of the herbs in all prescriptions. We focused on two parameters: support and confidence level. Support was set as ≥30% and confidence level as ≥85% [11] and a total of 30 herb pairs and suitable association rules were obtained. The detailed association rules are shown in Table 3, and the correlation rules are shown in Figure 3. As shown in Table 3, {Astragali Radix (Huangqi)}=>{Rhizoma Dioscoreae (Shanyao)} had the highest degree of support at 51.51%, {Rhizoma Atractylodis Macrocephalae (Baizhu), Fructus Corni (Shanzhuyu)}=>{Rhizoma Dioscoreae (Shanyao)} had the highest confidence level at 95.88%, and {Poria (Fulin), Radix Astragali (Huangqi)}=>{Rhizoma Atractylodis Macrocephalae (Baizhu)} had the highest degree of lift at 1.68.

**Figure 4:**
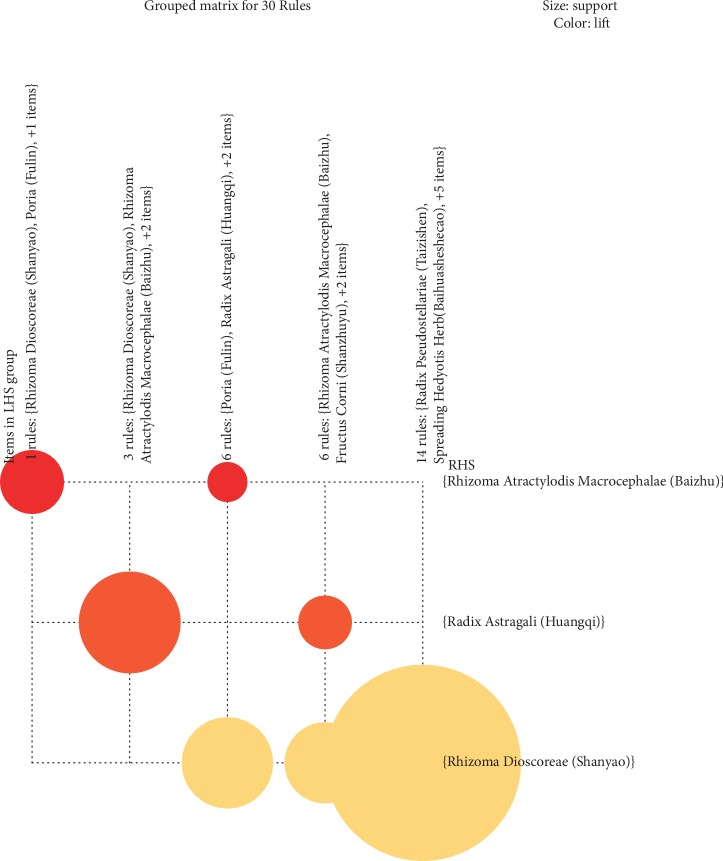
Association rule diagram. Association rule learning was performed using R-studio 3.5.3. *X-axis* is the antecedent (or called lefthandside, LHS) and *Y-axis* is the consequent (or called right-hand side, RHS). Size indicates support and color indicates lift.

A grouping matrix diagram displaying the general distribution of association rules grouped into categories with similar rules is shown in Figure 4. This diagram allows us to not only extract the general rule but also more deeply search for and extract important rules with commonality among the association rules. *X*-axis is the LHS and *Y*-axis is the RHS. The circle's color depth indicates the degree of lift, such that the darker the color, the higher the degree of lift. The size of the circle indicates the degree of support, such that the larger the circle, the higher the degree of support. We found three core herbs of paired prescriptions: Astragali Radix (Huangqi), Rhizoma Dioscoreae (Shanyao), and Rhizoma Atractylodis Macrocephalae (Baizhu). These herbs were usually combined with other herbs, such as Astragali Radix (Huangqi) paired with Fructus Corni (Shanzhuyu), Rhizoma Dioscoreae (Shanyao) paired with Spreading Hedyotis Herb (Baihuasheshecao), and Rhizoma Atractylodis Macrocephalae (Baizhu) paired with Poria (Fulin) to strengthen the spleen, tonify the kidney, and eliminate dampness-heat, respectively.

In Figure 3, association rules were determined with a graphics-based visualization technique using a vertex to represent herbs and relationships. The strength of the relationship is expressed in the color depth or the size of the vertex. The graph includes Rhizoma Dioscoreae (Shanyao), Spreading Hedyotis Herb (Baihuasheshecao), Root of Snow of June (Baimagu), Radix Astragali (Huangqi), Poria (Fulin), Rhizoma Atractylodis Macrocephalae (Baizhu), Radix Pseudostellariae (Taizishen), and Fructus Corni (Shanzhuyu). Among these eight herbs, Radix Astragali (Huangqi) and Rhizoma Atractylodis Macrocephalae (Baizhu) are at the center of the association rule graph.

#### 3.5.2. Novel Prescriptions Analysis

**Figure 5:**
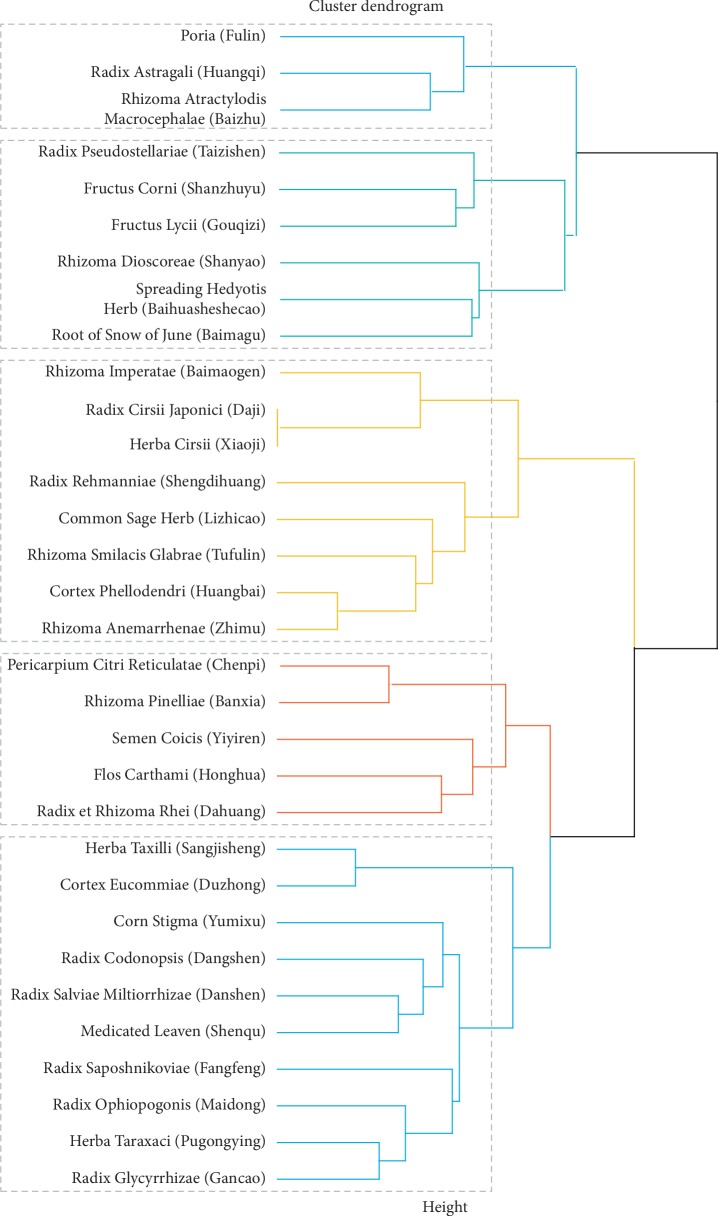
Cluster analysis tree diagram. The cluster analysis tree diagram was created using R-studio 3.5.3. The 32 most frequently prescribed herbs were analyzed. Each category is represented by a different color.

Cluster analysis aggregates herbs into categories based on data attributes. The clustering algorithm is an unsupervised learning algorithm that can directly extract valuable information from the input data objects without input of any prior knowledge. It is widely used for data mining in TCM and mainly used for determining the compatibility law between drugs to identify the combination rule of different TCM therapeutic methods. The advantage of this method is that it allows for discovery of potential new prescriptions for CKD treatment. Here, we analyzed 32 core herbs that were prescribed more than 30 times using hierarchical clustering. According to classic TCM theory, five categories were considered suitable in this study.  Figure 5 shows the herbs belonging to each cluster.

#### 3.5.3. Core Prescription Analysis

**Figure 6:**
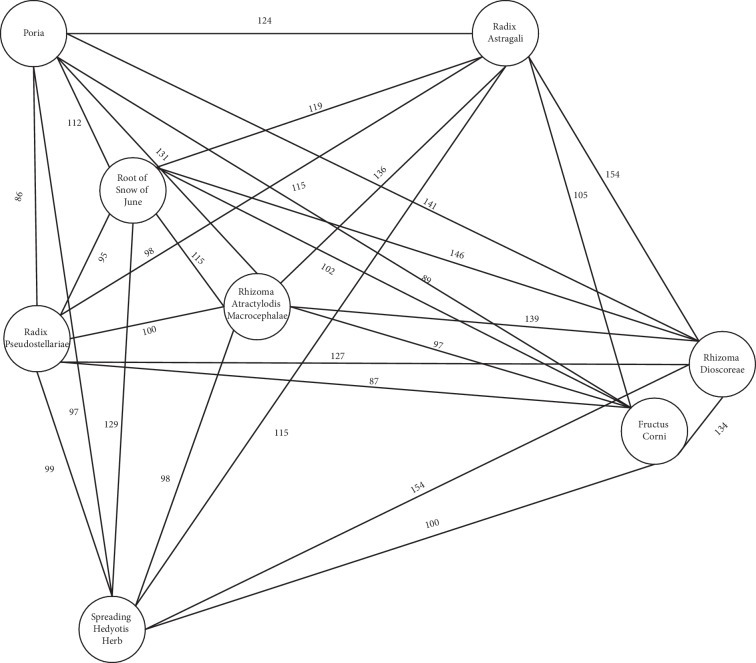
Core prescription network. The core prescription network was created using Liquorice. The weight represents the frequency with which two herbs appeared together.

Chinese medicinal herbs are usually applied as a combination of core herbs and other herbs. To further analyze the role of various herbs and their combinations in CKD treatment, we used the complex network method and found that the core herbs in CKD treatment were Rhizoma Dioscoreae (Shanyao), Spreading Hedyotis Herb (Baihuasheshecao), Root of Snow of June (Baimagu), Radix Astragali (Huangqi), Poria (Fulin), Rhizoma Atractylodis Macrocephalae (Baizhu), Radix Pseudostellariae (Taizishen), and Fructus Corni (Shanzhuyu) (Figure 6). These eight herbs were also found to be frequently prescribed for CKD treatment and appeared in the association rule graph. These results indicate that these Chinese medicinal herbs may have not only an effect on CKD treatment but also a united one.

## 4. Discussion

This study determined effective herbal prescriptions for CKD treatment by proposing a comprehensive method based on clinical cases, integration of association rules, cluster analysis, and complex network analysis.

We analyzed herb frequency and the properties and actions of herbs and assessed the compatibility rules between drugs. Our data showed that the most frequently prescribed herbs were Rhizoma Dioscoreae (Shanyao), Spreading Hedyotis Herb (Baihuasheshecao), Root of Snow of June (Baimagu), Radix Astragali (Huangqi), Poria (Fulin), Rhizoma Atractylodis Macrocephalae (Baizhu), Radix Pseudostellariae (Taizishen), Fructus Corni (Shanzhuyu), Radix Rehmanniae (Shengdihuang), and Fructus Lycii (Gouqizi). The properties of these herbs were mainly being warm, mild, and cold. The tastes of the herbs were mainly sweet and bitter. The herbs were generally distributed to spleen, lung, liver, and kidney channels, and most were deficiency-tonifying herbs, heat-clearing herbs, and dampness-draining diuretic herbs. The most frequently used combinations of herbs obtained using association rules were Radix Astragali (Huangqi) and Rhizoma Dioscoreae (Shanyao). There were three core herbs of paired prescriptions: Rhizoma Atractylodis Macrocephalae (Baizhu), Radix Astragali (Huangqi), and Rhizoma Dioscoreae (Shanyao). Cluster analysis was used to divide the herbs into five clusters. Herbs in cluster 1 mainly targeted the spleen and lung, herbs in cluster 2 were prescribed to nourish the liver-kidney and to clear heat-dampness, and herbs in cluster 3 were prescribed for clearing heat, cooling blood, and clearing dampness. Cluster 4 herbs were prescribed for regulating qi flow, eliminating phlegm, activating blood, and purging turbidity. Herbs in cluster 5 were not frequently used. We used the complex network method to analyze interactions between herbs and found that the core herbs were Rhizoma Dioscoreae (Shanyao), Spreading Hedyotis Herb (Baihuasheshecao), Root of Snow of June (Baimagu), Radix Astragali (Huangqi), Poria (Fulin), Rhizoma Atractylodis Macrocephalae (Baizhu), Radix Pseudostellariae (Taizishen), and Fructus Corni (Shanzhuyu).

The properties and actions of Chinese medicinal herbs are the essential bases for the analysis and clinical use of herbs. They are summarized in medical practice and based on the theories of yin and yang, zang-fu, meridian tropisms, and TCM therapeutic principles [17]. Chinese medicinal herb theory is usually summarized as four properties and five tastes, meridian tropism, and so on. In this study, the herbal properties for CKD treatment were mainly sweet and warm, which have a replenishing effect. The next most frequently prescribed herbs had bitter and cold properties, which generally have a clearing action. Thus, tonifying deficiency was the most important CKD treatment, followed by clearing and disinhibiting. This finding reflects the concept that TCM replenishes missing substances and rids the body of excess substances to regulate the balance of the body. Meridian tropism is a theory that helps in understanding the orientation of drug action. In this study, the herbs were generally prescribed to target the spleen, liver, lung, stomach, and kidney meridian (i.e., the zang-fu requiring treatment). In one study's retrospective analysis, the basic syndromes of kidney disease were identified as spleen and kidney deficiency, lung and kidney qi deficiency, and liver and kidney yin deficiency [18]. Our research further indicates the important role of the kidney, spleen, stomach, liver, and lung in nephropathy. Similarly, the efficacious herbs were mainly deficiency-tonifying herbs, heat-clearing herbs, and dampness-draining diuretic herbs. Thus, these were the main TCM treatments for CKD. Conversely, the therapeutic principle could be used to determine the pathogenesis of the disease. That is, the pathogenic factors could be divided into heat and dampness and deficiency may be mainly caused by the kidney, spleen, lung, and liver.

We identified commonly used herbs and their combinations (Tables 1 and 3). The main herbs for CKD treatment were Rhizoma Dioscoreae (Shanyao), Radix Astragali (Huangqi), Poria (Fulin), Rhizoma Atractylodis Macrocephalae (Baizhu), Radix Pseudostellariae (Taizishen), and Fructus Corni (Shanzhuyu); these all have the function of replenishing. Specifically, Rhizoma Dioscoreae (Shanyao) targets the spleen, lung, and kidney. Radix Astragali (Huangqi) and Radix Pseudostellariae (Taizishen) can supplement the lung and spleen. Macrocephalae (Baizhu) and Poria (Fulin) are prescribed to invigorate the spleen, and Fructus Corni (Shanzhuyu) can nourish the liver and kidney. These six drugs in different combinations comprised the 11 most frequent combinations. Among them, the most frequent combinations included Rhizoma Dioscoreae (Shanyao). Rhizoma Dioscoreae (Shanyao) can treat all deficiency syndromes according to TCM theory and is important for tonifying deficiency. Thus, herb combinations showed that the tonifying-deficiency category was the most important for CKD treatment. In TCM nephrology, kidney deficiency is the basis of kidney disease and tonifying kidney treatment should be vital [19]. However, our research showed that the methods of supplementing the lung and spleen and nourishing the liver and kidney were more frequently used in CKD. Tonifying the spleen, lung, or liver is well known to achieve the purpose of nourishing the kidney but herbs that tonify the kidney were not frequently prescribed in this research. This indicates that a multiple-organ disorder underlies the TCM pathogenesis of CKD. When and how to nourish the kidney directly or indirectly requires further study. In TCM practice, tonifying-deficiency herbs are often used in combination with heat-clearing and dampness-draining diuretic herbs. Table 3 shows that two or more herbs were usually combined with Spreading Hedyotis Herb (Baihuasheshecao) or Root of Snow of June (Baimagu), which were the two most frequently used heat-clearing herbs and dampness-removing diuretic herbs. Tonifying treatment was also indicated to be more important than heat-clearing or dampness-draining treatment. How to balance tonifying treatment and clearing-diuresis treatment under specific conditions needs to be investigated further.

The pathogenesis of kidney disease is complex and often manifests as multiple syndrome combinations. The formulae determined by clustering were for composite syndromes. Herbs in cluster 1 were for tonifying the lung, strengthening the spleen, and removing dampness and were prescribed to treat spleen-lung qi deficiency and dampness. Herbs in cluster 2 were for nourishing the liver and kidney and clearing heat or dampness and were prescribed to treat liver-kidney yin deficiency and dampness or heat. Those in cluster 3 were for nourishing yin, clearing heat, and cooling blood. These herbs can be found in Zhi Bai Di Huang Wan and are prescribed to treat fire excess from yin deficiency and blood-heat syndrome. Those in cluster 4 were for regulating qi flow, eliminating phlegm, activating blood, and purging turbidity. They were prescribed to treat phlegm-damp, blood stasis, and turbidity syndromes. These four clusters of prescriptions for different syndrome types can provide useful ideas for CKD treatment. The herbs in cluster 5 had multiple functions, such as tonifying the kidney, releasing the exterior, and promoting digestion. It may be used auxiliarily to target complications and requires further study.

Complex network analysis revealed eight core herbs for CKD: Rhizoma Dioscoreae (Shanyao), Spreading Hedyotis Herb (Baihuasheshecao), Root of Snow of June (Baimagu), Radix Astragali (Huangqi), Poria (Fulin), Rhizoma Atractylodis Macrocephalae (Baizhu), Radix Pseudostellariae (Taizishen), and Fructus Corni (Shanzhuyu). Interestingly, these herbs are included in clusters 1 and 2. They were also identified as high-frequency herbs for CKD treatment, so we believed that such a prescription could be used as a basis for further research. Modern pharmacology has also shown that these herbs can improve kidney injury through anti-inflammatory and antioxidant activity (Table 4) [20–29].

Our data mining approach has several advantages. First, there was no requirement for the data structure. This is very practical for data mining of Chinese medicine prescriptions because the data structure of most Chinese medicines is not uniform. Second, we combined a variety of data mining methods to perform comprehensive analysis and to ensure that the conclusion was reliable. Finally, we focused on the main treatment methods and uses of herbs, which is an efficient way to learn TCM treatment. Our research also has some limitations. We collected only prescription information but not diagnostic information, so our conclusions cannot be completely confirmed. We also examined only effective treatment cases, not invalid treatment cases, so whether a gap exists between these two case types remains unclear. Thus, it is also unclear whether the core prescription should be corrected. Finally, the safety and efficacy of the core prescriptions were not evaluated and should be investigated in a future study.

In summary, we adopted a practical approach based on formulae prescribed in clinical practice. To our knowledge, this is the first study on CKD treatment with TCM which combines frequency analysis, association rule learning, and complex network analysis. Using this approach, we determined the frequency and combination patterns of CKD treatment and discovered possibilities for new CKD treatment prescriptions. We also summarized herbal CKD treatment, finding that CKD syndromes are extremely complicated and various CKD treatment methods exist, such as those for cooling blood and eliminating phlegm. However, we found that the treatment methods were mainly for tonifying deficiency and clearing heat or dampness.

## 5. Conclusions

Our study found that kidney, spleen, lung, or liver deficiency and dampness-heat were the primary TCM pathogenesis. Thus, the primary treatment principle was tonifying deficiency and eliminating dampness and heat. Furthermore, our study verified that the integrated analysis method can help to explore the TCM treatment strategy. This analysis of herbal TCM prescriptions potentially contributes to the development of novel drugs for CKD.

## Figures and Tables

**Table 1:** Herbs appearing over 40 times in prescriptions.

Herb	Number	Frequency
Rhizoma Dioscoreae (Shanyao)	231	0.773
Spreading Hedyotis Herb (Baihuasheshecao)	196	0.656
Root of Snow of June (Baimagu)	175	0.585
Radix Astragali (Huangqi)	174	0.582
Poria (Fulin)	166	0.555
Rhizoma Atractylodis Macrocephalae (Baizhu)	162	0.542
Radix Pseudostellariae (Taizishen)	143	0.478
Fructus Corni (Shanzhuyu)	140	0.468
Radix Rehmanniae (Shengdihuang)	102	0.341
Fructus Lycii (Gouqizi)	100	0.334
Flos Carthami (Honghua)	96	0.321
Common Sage Herb (Lizhicao)	86	0.288
Pericarpium Citri Reticulatae (Chenpi)	84	0.281
Cortex Phellodendri (Huangbai)	83	0.278
Semen Coicis (Yiyiren)	80	0.268
Rhizoma Imperatae (Baimaogen)	79	0.264
Rhizoma Anemarrhenae (Zhimu)	78	0.261

**Table 2:** Frequency of herb categories.

Herb category	Frequency	Rate (%)
Deficiency-tonifying herbs	1233	29.91
Heat-clearing herbs	1027	24.91
Dampness-draining diuretic herbs	410	9.94
Hemostatic herbs	198	4.8
Astringent herbs	170	4.12
Qi-regulating herbs	169	4.1
Blood-activating and stasis-resolving herbs	162	3.93
Cough-suppressing and panting-calming herbs	151	3.66
Exterior-releasing herbs	134	3.25
Digestant herbs	115	2.79
Wind-dampness dispelling herbs	94	2.28
Dampness-resolving medicine	83	2.01
Purgating drug	70	1.7
Nerve-soothing herbs	55	1.33
Interior-warming herbs	27	0.65
Liver-wind calming herbs	24	0.58
Antitoxin, insecticide, and antipruritic	1	0.02

**Table 3:** Association rules of herbs for CKD treatment.

Items (LHS=>RHS)	Support (%)	Confidence (%)	Lift
{Radix Astragali (Huangqi)}=>{Rhizoma Dioscoreae (Shanyao)}	51.51	88.51	1.15
{Poria (Fulin)}=>{Rhizoma Dioscoreae (Shanyao)}	47.16	85.45	1.11
{Rhizoma Atractylodis Macrocephalae (Baizhu)}=>{Rhizoma Dioscoreae (Shanyao)}	46.49	86.34	1.12
{Fructus Corni (Shanzhuyu)}=>{Rhizoma Dioscoreae (Shanyao)}	44.82	95.71	1.24
{Radix Pseudostellariae (Taizishen)}=>{Rhizoma Dioscoreae (Shanyao)}	42.14	88.73	1.15
{Rhizoma Atractylodis Macrocephalae (Baizhu), Radix Astragali (Huangqi)}=>{Rhizoma Dioscoreae (Shanyao)}	41.14	90.44	1.17
{Rhizoma Atractylodis Macrocephalae (Baizhu), Rhizoma Dioscoreae (Shanyao)}=>{Radix Astragali (Huangqi)}	41.14	88.49	1.52
{Rhizoma Atractylodis Macrocephalae (Baizhu), Poria (Fulin)}=>{Rhizoma Dioscoreae (Shanyao)}	39.13	89.31	1.16
{Poria (Fulin), Radix Astragali (Huangqi)}=>{Rhizoma Dioscoreae (Shanyao)}	38.13	91.94	1.19
{Rhizoma Atractylodis Macrocephalae (Baizhu), Poria (Fulin)}=>{Radix Astragali (Huangqi)}	37.46	85.50	1.47
{Poria (Fulin), Radix Astragali (Huangqi)}=>{Rhizoma Atractylodis Macrocephalae (Baizhu)}	37.46	90.32	1.68
{Root of Snow of June (Baimagu), Radix Astragali (Huangqi)}=>{Rhizoma Dioscoreae (Shanyao)}	36.45	91.60	1.19
{Radix Astragali (Huangqi), Radix Pseudostellariae (Taizishen)}=>{Rhizoma Dioscoreae (Shanyao)}	34.45	90.35	1.17
{Rhizoma Spreading Hedyotis Herb (Baihuasheshecao), Radix Astragali (Huangqi)}=>{Rhizoma Dioscoreae (Shanyao)}	34.45	89.57	1.16
{Rhizoma Atractylodis Macrocephalae (Baizhu), Poria (Fulin), Radix Astragali (Huangqi)}=>{Rhizoma Dioscoreae (Shanyao)}	34.11	91.07	1.18
{Rhizoma Atractylodis Macrocephalae (Baizhu), Poria (Fulin), Rhizoma Dioscoreae (Shanyao)}=>{Radix Astragali (Huangqi)}	34.11	87.18	1.50
{Poria (Fulin), Radix Astragali (Huangqi), Rhizoma Dioscoreae (Shanyao)}=>{Rhizoma Atractylodis Macrocephalae (Baizhu)}	34.11	89.47	1.66
{Root of Snow of June (Baimagu), Rhizoma Atractylodis Macrocephalae (Baizhu)}=>{Radix Astragali (Huangqi)}	33.78	87.83	1.51
{Root of Snow of June (Baimagu), Rhizoma Atractylodis Macrocephalae (Baizhu)}=>{Rhizoma Dioscoreae (Shanyao)}	33.78	87.83	1.14
{Radix Astragali (Huangqi), Fructus Corni (Shanzhuyu)}=>{Rhizoma Dioscoreae (Shanyao)}	33.44	95.24	1.23
{Root of Snow of June (Baimagu), Poria (Fulin)}=>{Rhizoma Dioscoreae (Shanyao)}	33.44	89.29	1.16
{Root of Snow of June (Baimagu), Fructus Corni (Shanzhuyu)}=>{Rhizoma Dioscoreae (Shanyao)}	32.44	95.10	1.23
{Root of Snow of June (Baimagu), Poria (Fulin)}=>{Rhizoma Atractylodis Macrocephalae (Baizhu)}	32.44	86.61	1.61
{Rhizoma Spreading Hedyotis Herb (Baihuasheshecao), Fructus Corni (Shanzhuyu)}=>{Rhizoma Dioscoreae (Shanyao)}	31.44	94.95	1.23
{Rhizoma Atractylodis Macrocephalae (Baizhu), Fructus Corni (Shanzhuyu)}=>{Rhizoma Dioscoreae (Shanyao)}	31.10	95.88	1.24
{Root of Snow of June (Baimagu), Rhizoma Atractylodis Macrocephalae (Baizhu), Radix Astragali (Huangqi)}=>{Rhizoma Dioscoreae (Shanyao)}	30.77	91.09	1.18
{Root of Snow of June (Baimagu), Rhizoma Atractylodis Macrocephalae (Baizhu), Rhizoma Dioscoreae (Shanyao)}=>{Radix Astragali (Huangqi)}	30.77	91.09	1.57
{Rhizoma Atractylodis Macrocephalae (Baizhu), Radix Pseudostellariae (Taizishen)}=>{Rhizoma Dioscoreae (Shanyao)}	30.43	91.00	1.18
{Rhizoma Atractylodis Macrocephalae (Baizhu), Fructus Corni (Shanzhuyu)}=>{Radix Astragali (Huangqi)}	30.10	92.78	1.59
{Radix Astragali (Huangqi), Fructus Corni (Shanzhuyu)}=>{Rhizoma Atractylodis Macrocephalae (Baizhu)}	30.10	85.71	1.59

**Table 4:** Possible mechanisms of the core Chinese medical herbs for CKD treatment.

Chinese herbs	Active ingredients	Mechanism
Rhizoma Dioscoreae (Shanyao)	Dioscin	Adjusts oxidative stress, fibrosis, lipid metabolism, and inflammation against renal damage [20, 21].
Spreading Hedyotis Herb (Baihuasheshecao)	Water extract	Suppresses the productions of tumor necrosis factor-*α* (TNF-*α*), interleukin-1*β* (IL-1*β*), IL-6, and monocyte chemoattractant protein-1 (MCP-1), as well as promoting the production of IL-10 in serum and renal tissue [22].
Root of Snow of June (Baimagu)		Improves the ability to remove antigens, restore the glomerular basement membrane, and increase the renal blood flow [23].
Radix Astragali (Huangqi)	Astragaloside IV	Immunomodulatory, antioxidative, and anti-inflammatory [24, 25].
Rhizoma Atractylodis Macrocephalae (Baizhu)	Polysaccharide	Decreases the productions of IL-6 and TNF-*α*, increase the level of superoxide dismutase (SOD), and improve the renal tissue injury [26].
Poria (Fulin)	Lanostane triterpenoids	Inhibits JNK, ERK, p38, and caspase-3 against cisplatin-induced kidney tubular epithelial cells injury [27].
Fructus Corni (Shanzhuyu)	Ethanol extract	Increases catalase (CAT), superoxide dismutase (SOD), and glutathione peroxidase (GSH-px) activities in the kidneys of diabetic rats as well as enhancing renal peroxisome proliferator-activated receptor-*γ* (PPAR*γ*) expression in diabetic rats [28].
Radix Pseudostellariae (Taizishen)	Polysaccharide	Decreases serum triglyceride, total cholesterol, low-density-lipoprotein cholesterin, urea nitrogen, and creatinine, increase serum high-density-lipoprotein cholesterol, and reduce renal histopathology change [29].

## Data Availability

The data used to support the findings of this study are included within the article.
